# Phenotypic characterization of Ghanaian *P. falciparum* clinical isolates reveals a homogenous parasite population

**DOI:** 10.3389/fimmu.2022.1009252

**Published:** 2022-09-23

**Authors:** Laty G. Thiam, Prince B. Nyarko, Felix Ansah, Makhtar Niang, Gordon A. Awandare, Yaw Aniweh

**Affiliations:** ^1^ West African Centre for Cell Biology of Infectious Pathogens, College of Basic and Applied Sciences, University of Ghana, Accra, Ghana; ^2^ Department of Biochemistry Cell and Molecular Biology, College of Basic and Applied Sciences, University of Ghana, Accra, Ghana; ^3^ Pôle Immunophysiopathologie et Maladies Infectieuses, Institut Pasteur de Dakar, Dakar, Senegal

**Keywords:** merozoites, clinical isolates, phenotype, invasion, neutralizing

## Abstract

**Background:**

Erythrocyte invasion by P. falciparum involves functionally overlapping interactions between the parasite’s ligands and the erythrocyte surface receptors. While some P. falciparum isolates necessarily engage the sialic acid (SA) moieties of the erythrocytes during the invasion, others use ligands whose binding is independent of SA for successful invasion. Deciphering the major pathway used by P. falciparum clinical isolates represent a key step toward developing an efficient blood stage malaria vaccine.

**Methods:**

We collected a total of 156 malaria-infected samples from Ghanaian children aged 2 to 14 years and used a two-color flow cytometry-based invasion assay to assess the invasion phenotype diversity of Ghanaian P. falciparum clinical isolates. Anti-human CR1 antibodies were used to determine the relative contribution of the PfRh4-CR1 interaction in the parasites invasion phenotype and RT-qPCR was used to assess the expression levels of key invasion-related ligands.

**Results:**

Our findings show no clear association between demographic or clinical data and existing reports on the malaria transmission intensity. The complete invasion data obtained for 156 isolates, showed the predominance of SA-independent pathways in Ghanaian clinical isolates. Isolates from Hohoe and Navrongo had the highest diversity in invasion profile. Our data also confirmed that the PfRh4-CR1 mediated alternative pathway is important in Ghanaian clinical isolates. Furthermore, the transcript levels of ten invasion-related genes obtained in the study showed little variations in gene expression profiles within and between parasite populations across sites.

**Conclusion:**

Our data suggest a low level of phenotypic diversity in Ghanaian clinical isolates across areas of varying endemicity and further highlight its importance in the quest for new intervention strategies, such as the investigation of blood-stage vaccine targets, particularly those targeting specific pathways and able to trigger the stimulation of broadly neutralizing invasion antibodies.

## Introduction

Despite tremendous efforts that led to the drastic reduction of the malaria burden (1), *Plasmodium falciparum* malaria continues to be a major public health concern, claiming more than 400,000 with the sub-Saharan region of Africa being the most afflicted worldwide (2). Thus, the development of an affordable, yet efficacious malaria vaccine remains one of the last resorts to achieve the goal of elimination. The clinical manifestations of malaria only occur during the asexual replication of the parasites in the host erythrocytes. Likewise, development of the sexual transmissive forms occurs at the blood stage. Consequently, the blood stage, particularly erythrocyte invasion, represents an attractive interventional target.

Erythrocyte invasion by *P. falciparum* is a very rapid and complex process involving the sequential release of an arsenal of parasite antigens (ligands) from the merozoite’s secretory organelles (3), which subsequently bind to specific receptors on the surface of the host erythrocyte to mediate invasion (4). *P. falciparum* invasion-related ligands include members of the *P. falciparum* erythrocyte binding antigens/ligands (PfEBAs/PfEBLs), namely, PfEBA140, PfEBA165, PfEBA175, PfEBA181 and PfEBL1, as well as members of the *P. falciparum* reticulocyte binding-like homologue (PfRh) family proteins, including PfRh1, PfRh2a, PfRh2b, PfRh4 and PfRh5 (5). Cumulatively, PfEBA and PfRh proteins are essential for the survival of the parasite. However, with the exception of PfRh5, these ligands appear to be functionally redundant (5). While some of these ligands bind to sialic acids (SA) on erythrocyte surface glycoproteins for successful invasion by some strains, others bind erythrocytes and mediate invasion independent of SA. Consequently, *P. falciparum* invasion pathways are broadly classified as either SA-dependent or SA-independent. Of the five *P. falciparum* ligand-receptor interactions known to be involved in invasion, three mediate the SA-dependent pathway (PfEBA175/glycophorin A, PfEBL1/glycophorin B and PfEBA140/glycophorin C), whereas the PfRh4/complement receptor 1 and the PfRh5/basigin mediate the SA-independent pathway (4, 6). *P. falciparum* uses the mechanism of phenotypic variation to circumvent host immune response during malaria infection PMC5893586 and PMCID: PMC6959351 (7–9). Therefore, characterizing the phenotypic invasion diversity of *P. falciparum* clinical isolates would provide relevant data with regards to the major invasion pathways used by these parasites during natural infections. This in turn could be exploited as a key resource in the quest for an effective malaria vaccine.

Pioneering studies on the invasion diversity of *P. falciparum* clinical isolates reported distinct repetitive pathways used by isolates from different geographical locations. For instance, clinical isolates from both The Gambia (10) and Brazil (11) have been shown to preferentially use the SA-dependent pathway, while isolates from India (12), Ghana (13), and Senegal (14, 15) predominantly use the SA-independent pathway. This led to the hypothesis that *P. falciparum* phenotypic diversity is influenced by the level of naturally acquired immunity against the circulating parasite populations, which in itself is largely influenced by transmission intensities (6, 13). Previous reports from our group showed that *P. falciparum* clinical isolates from three endemic areas in Ghana predominantly used SA-independent invasion pathway. However, while sensitivity to neuraminidase treatment was inversely proportional to transmission intensity, such trend was not observed upon treatment with trypsin or chymotrypsin (13). In this study, we included a larger number of *P. falciparum* clinical isolates from four endemic areas in Ghana to assess their phenotypic invasion diversity. Given the earlier reports on the use of complement receptor 1 (CR1) as a key receptor for invasion in Ghanaian clinical isolates (6, 13); we also tested the contribution of the CR1-mediated pathway in our study population. Furthermore, we tested for the differential expression of invasion-related genes within the PfEBA and PfRh family proteins in selected isolates from our study sites.

## Materials and methods

### Sample collection

This study was approved by Ethics Committees of the Ghana Health Service, the Kintampo and Navrongo Health Research Centres and the Noguchi Memorial Institute for Medical Research. *P. falciparum* clinical isolates used in this study were collected from children aged 2 to 14 years, presenting clinically diagnosed malaria infection, visiting the LEKMA Hospital (Accra), the Hohoe Municipal Hospital (Hohoe), the Kintampo Health Research Centre (Kintampo) or the Navrongo Health Research Centre (Navrongo). According to the most recent data regarding transmission intensities, Kintampo has the highest transmission rate with an estimated entomological inoculation rate (EIR) of >250 infective mosquito bites per person/year (16), followed by Navrongo with <250 infective mosquito bites per person/year (17), while Hohoe (18) and Accra (19) have the lowest transmission rates with 65 and <50 infective mosquito bites per person/year, respectively. For all participants, written consent was obtained from the parent or guardian before sample collection and additional assent was sought from children older than 10 years. *P. falciparum* infections were confirmed by light microscopy of Giemsa stained thin and thick films following a positive rapid diagnostic test. The parasitemia was estimated as previously described (13). Samples were collected in acid citrate dextrose vacutainers and parasitized erythrocytes were cryopreserved with glycerolyte in liquid nitrogen until use.

### *In vitro* culturing and invasion assays

*P. falciparum* clinical isolates were cultured at 4% hematocrit in complete parasite medium (RPMI 1640 containing 25 mM HEPES, 0.5% Albumax II, 2 mg/mL sodium bicarbonate and 50 μg/mL Gentamicin and 2% normal human serum) (PMCID:PMC8007732) and incubated at 37° C in an atmosphere of 2% O_2_, 5% CO_2_ and balanced with N_2_. Human erythrocytes of blood group O^+^ were used for all cultures and invasion assays. Erythrocytes used in invasion assays were treated with either neuraminidase (250 mU/mL), trypsin (1 mg/mL) or chymotrypsin (1 mg/mL) for an hour at 37° C with gentle shaking and further labelled with 20 µM carboxyfluorescein diacetate succinimidyl ester (CFDA-SE) for two hours at 37° C. Parasites were ex-vivo cultured for at least 36 hours and invasion assays were set as previously described (13, 20). Additionally, to determine the relative dependence of clinical isolates on the CR1-mediated pathway, assays were set in the presence of 12 µg/mL of chicken anti-human CR1 antibodies or isotype control immunoglobulin Y (IgY, Gallus Immunotech, Fergus, Canada). Culture-derived infected erythrocytes were seeded with equal volumes of target labelled erythrocytes and incubated at 37° C for approximately 24 hours. Plates were subsequently removed from the incubator and spun at 2000 rpm for 3 minutes and the medium replaced with 5 µM Hoechst 33342 solution to label the parasites’ DNA. Invasion rates were determined using a BD LSR Fortessa X-20 cytometer (BD Biosciences, Belgium).

### Gene expression analysis

The All-Prep DNA/RNA Mini Kit (Qiagen) was used to extract total RNAs from schizont-infected erythrocytes, and the Luna Universal One-Step RT qPCR Kit (New England Biolabs, Inc.) was used to assess the expression level of selected invasion-related genes. For each isolate, the transcript level of individual genes was determined as the percentage of the sum of transcript levels of all genes after normalization with the 60S ribosomal protein L18 and the apical membrane antigen 1 (AMA1) as endogenous control and parasites maturation marker, respectively

### Statistical analysis

All statistical analyses were performed using GraphPad Prism v.8.01. The Chi-square test was used to compute for differences between categorical variables. One-way ANOVA or Kruskal Wallis was used to assess for differences between distinct groups and where significant, the Tukey’s or the Dunn’s multiple comparison test was used for pairwise comparisons, respectively. The Spearman rank correlation or the Pearson correlation was used to assessing relationships between variables. For all analysis, a *p-value* of at least 0.05 was considered as significant.

## Results

### Characteristics of study participants

Participants for this study were children aged 2 to 14 years, presenting symptomatic *P. falciparum* infection, recruited from areas of varying transmission intensities; Accra (N = 42) < Hohoe (N = 36) < Navrongo (N = 38) < Kintampo (N = 40). The demographic and hematological characteristics of the study participants are summarized in **Table 1**. Overall, there was no significant difference in terms of sex ratio across the four study sites (Chi-square, P = 0.97). However, there was a significant difference in the mean age (ANOVA, P = 0.008), with children from Navrongo being the youngest, while the oldest children were from Hohoe. In respect of parasite density, children from Kintampo reported the highest parasite burden, while those from Navrongo were found to harbor the lowest number of parasites. There was a significant difference in the parasite burden in children from Kintampo compared to those from Accra (P *=* 0.002), Hohoe (P *=* 0.0003) and Navrongo (P *=* 0.0001), while no significant difference was observed among the three other sites. Furthermore, no significant variation was observed with the hemoglobin levels (**Table 1**).

**Table 1:** Clinical and demographic data of study participants from Accra, Hohoe, Navrongo and Kintampo.

Characteristics	Accra	Hohoe	Navrongo	Kintampo	Overall	*P-value*
**Donors, No.**	42	36	38	40	156	
**Demographic data**						
**Sex ratio (Male/Female)**	1.25	1.18	1.19	1.44	1.26	*0.97 ^a^*
**Age (Years), Mean ± SEM,**	5.75 ± 0.54	7 ± 0.56	4.67 ± 0.46	4.89 ± 0.49	5.58 ± 0.26	*0.008 ^a^*
**Haematological indices**						
**Parasite density (Parasites/uL), Mean ± SEM,**	69872 ± 7866	58333 ± 7988	52170 ± 4966	144811 ± 25688	82285 ± 7863	*<0.0001 ^b^*
**Hemoglobin level (g/dL), Mean ± SEM**	10.31 ± 0.29	10.79 ± 0.17	10.35 ± 0.29	9.70 ± 0.31	10.28 ± 0.14	*0.051 ^b^*

SEM: standard error of the mean.

^a^indicates test performed using Chi-square.

^b^indicates analysis performed with One-way ANOVA.

### Invasion pathways of Ghanaian *P. falciparum* clinical isolates

**Figure 1:**
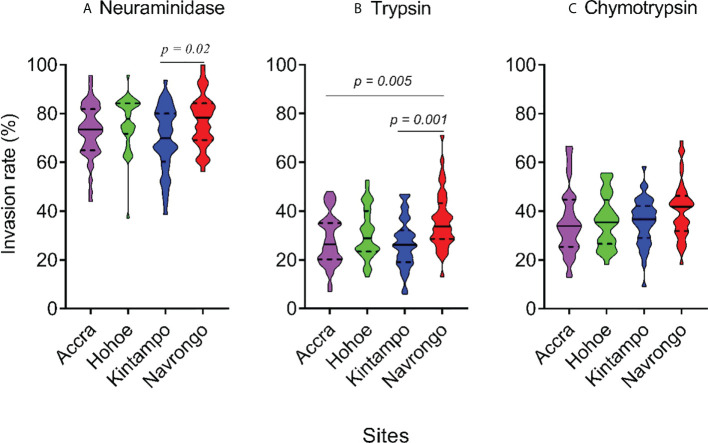
Violin plots showing the invasion phenotypes of *P. falciparum* clinical isolates. Different parasite isolates from areas of varying transmission intensities; Accra (N = 42) < Hohoe (N = 36) < Navrongo (N = 38) < Kintampo (N = 40) were tested. Invasion efficiency was determined by the parasite invasion efficiency into neuraminidase **(A)**, trypsin **(B)** or chymotrypsin **(C)** treated erythrocytes relative to the untreated control. Pairwise comparisons computed using the Tukey’s multiple comparison test showed differences in the invasion efficiency of parasites from Navrongo and Kintampo following neuraminidase treatment (*P* = 0.02) and between Navrongo and Accra or Kintampo following trypsin treatment (*P* = 0.005 and *P* = 0.001, respectively).

**Figure 2:**
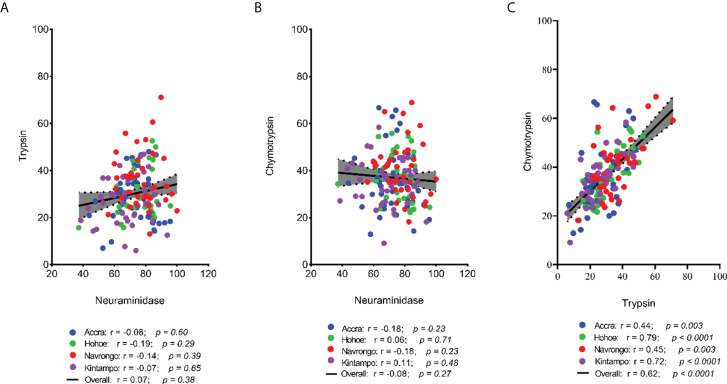
Scatter plots showing the pairwise correlations of the parasite invasion efficiency into enzyme-treated erythrocytes. The Pearson correlation was used to compute the coefficients of correlation between invasion efficiency into **(A)** neuraminidase and trypsin treated erythrocytes, **(B)** neuraminidase and chymotrypsin treated erythrocyte, and **(C)** trypsin and chymotrypsin treated erythrocytes.

To investigate the major invasion pathways used by Ghanaian *P. falciparum* clinical isolates, parasites were first tested for their propensity to invade enzyme-treated erythrocytes. Target erythrocytes were treated with either neuraminidase, which removes the SA residues of glycoproteins, trypsin, or chymotrypsin, which cleave the peptide backbones of receptors such as GYPA, GYPB, GYPC and CR1 (**Figure 1**). Across all sites, the invasion rate into neuraminidase-treated erythrocytes varied from 37.14 to 100% relative to untreated cells. Overall, the mean invasion rate into neuraminidase-treated erythrocytes was 74.85%, confirming earlier reports on the predominance of the SA-independent pathway in Ghanaian *P. falciparum* clinical isolates. Expectedly, there were differences in the invasion rate into neuraminidase-treated cells when comparing isolates across the different study sites (**Figure 1A**). Isolates from Navrongo showed the highest invasion rates into neuraminidase treated cells as compared to the rest of the study sites. However, this variation was only significant when comparing between isolates from Kintampo and Navrongo (P=0.02, **Figure 1A**). There was also wide variation in invasion rates following trypsin (mean, 28.92%) and chymotrypsin (mean, 35.87%) treatment, ranging from 5.97 to 71.11% and 9.09 to 68.89%, respectively (**Figures 1B, C**). Overall, isolates from Navrongo were less sensitive to treatment with trypsin or chymotrypsin. There was a significant difference in the invasion into trypsin-treated cells only when comparing isolates from Navrongo to those from Accra (P *=* 0.001, **Figure 1B**) or Kintampo (P = 0.005, **Figure 1B**). However, no significant difference was observed in the invasion into chymotrypsin treated erythrocytes across all study sites (P = 0.15, **Figure 1C**). Furthermore, there was no correlation between invasion into neuraminidase-treated erythrocytes with invasion into trypsin or chymotrypsin-treated erythrocytes (**Figures 2A, B**). However, there was a moderate to strong positive correlation in invasion efficiency between trypsin and chymotrypsin-treated erythrocytes across all sites (**Figure 2C**).

### Phenotypic diversity of Ghanaian *P. falciparum* clinical isolates

**Figure 3:**
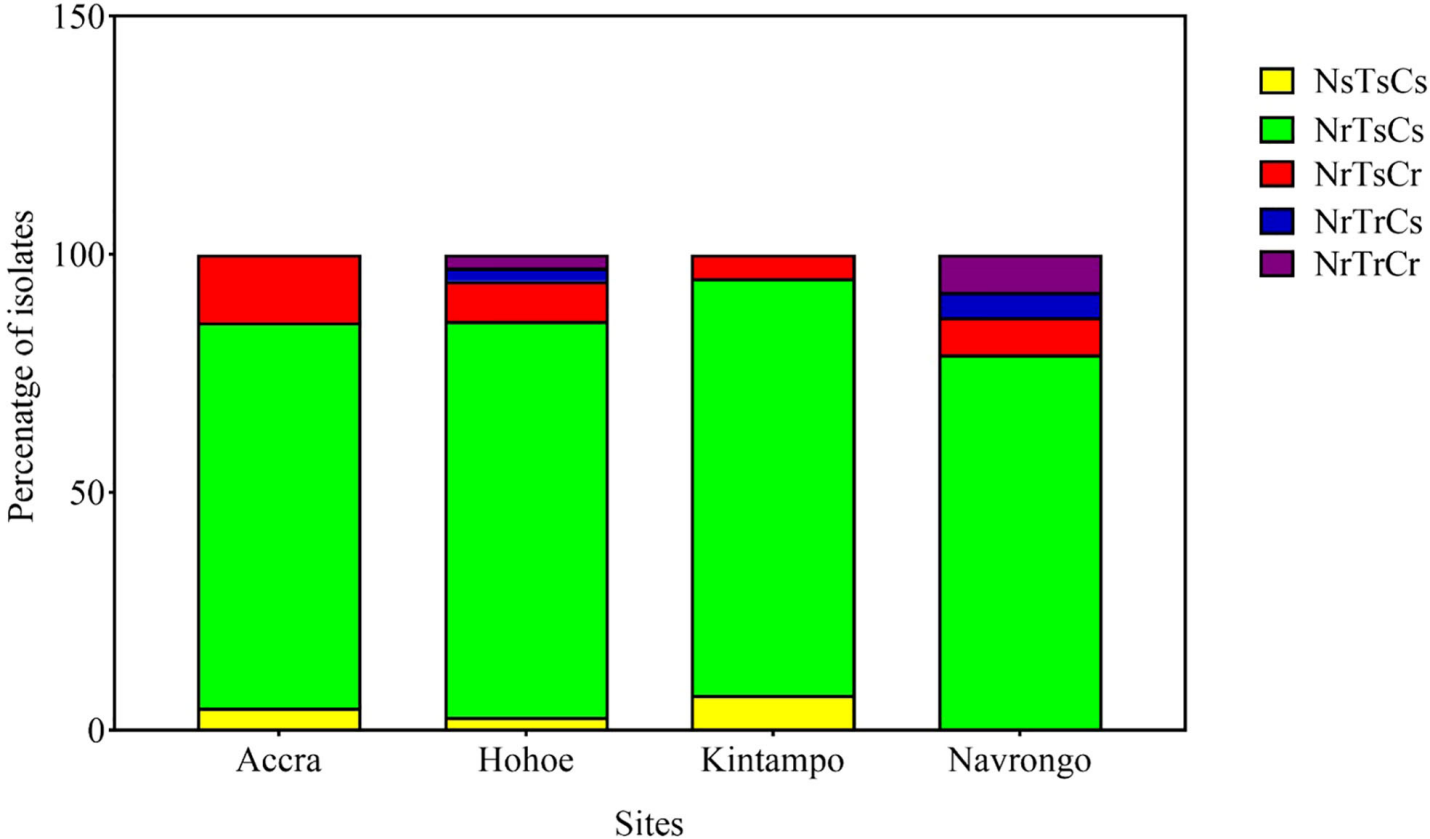
Invasion profiles of Ghanaian *P. falciparum* clinical isolates for areas of varying transmission intensity. The graph shows the proportion of a given profile within the parasite population from a given area. For each isolate, the invasion profile was determined as the combination of sensitivity to the three enzymes using a cut-off of 50% as a threshold to classify the observed profile as sensitive (≤50%) or resistance (>50%) for each given treatment. Consequently, isolates were classified as sensitive (s, <50% invasion) or resistant (r, >50% invasion) to neuraminidase (N), trypsin (T) or chymotrypsin (C).

Here, we defined the parasites’ invasion profile as the combined effect of the three enzymes (21). This was used to assess the phenotypic diversity of our isolates as a result of their invasion efficiency in enzyme-treated erythrocytes. A cut-off of 50% invasion efficiency relative to control erythrocytes was used to define sensitivity to a given treatment. Consequently, isolates were classified as sensitive (s, <50% invasion) or resistant (r, >50% invasion) to neuraminidase (N), trypsin (T) or chymotrypsin (C). Overall, five different profiles were observed across all sites, namely NsTsCs, NrTsCs, NrTsCr, NrTrCs and NrTrCr (**Figure 3**). Isolates from Hohoe and Navrongo presented the highest diversity with five and four different profiles, respectively (**Figure 3**). Across all sites, the NrTrCs and NrTrCr profiles were the least common and only present in Hohoe and Navrongo, while the NrTsCs profile was the commonest, present in all study sites (**Figure 3**). Moreover, there was no profile associated with malaria transmission intensity, agreeing with our previous findings (6).

### CR1 is a major receptor mediating SA-independent pathway in Ghanaian *P. falciparum* clinical isolates

**Figure 4:**
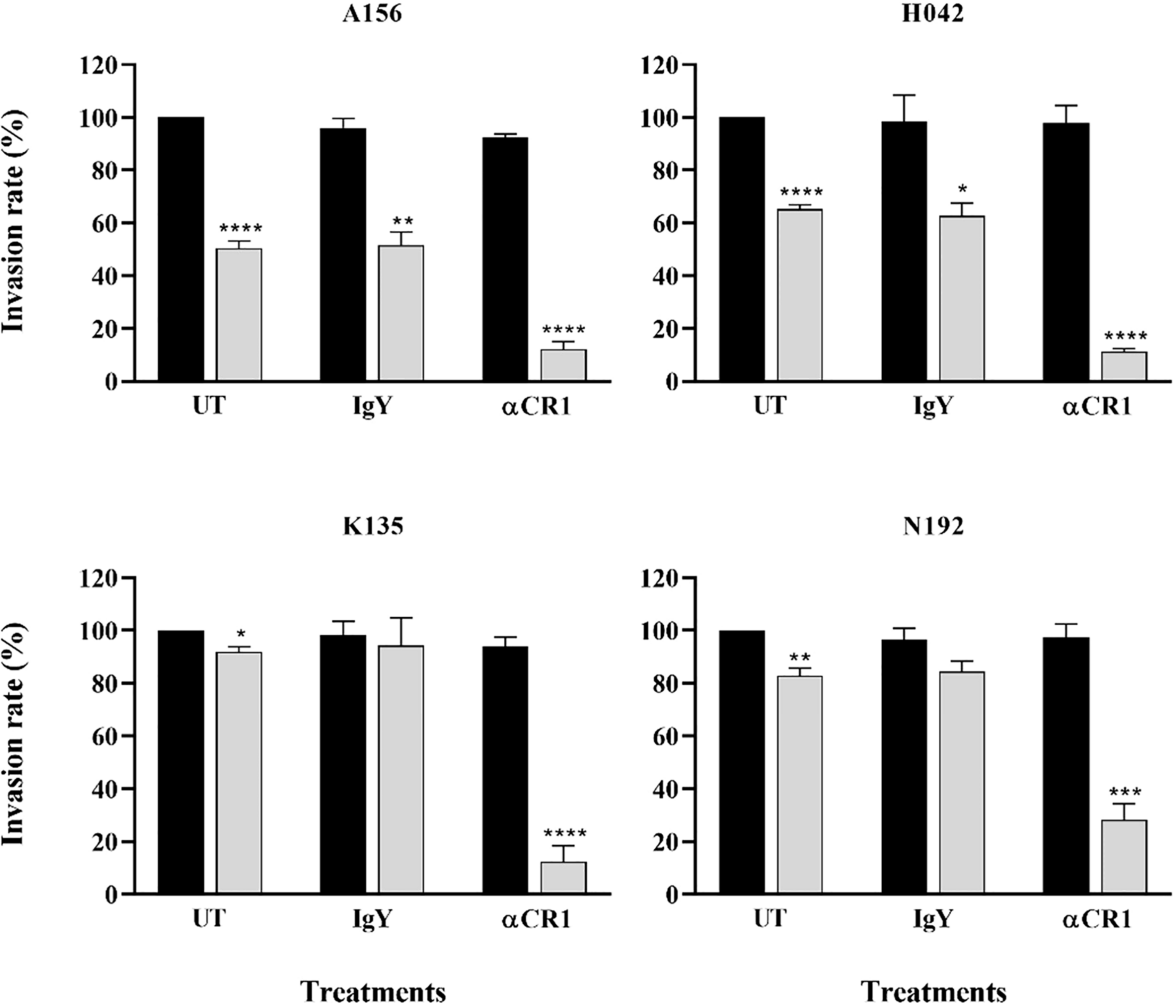
Representative graphs showing the importance of CR1 as an alternative receptor for invasion by Ghanaian *P. falciparum* clinical isolates. Invasion efficiency of different parasite isolates (A156, H042, K135 and N192) into untreated (black bars) and neuraminidase treated (gray bars) erythrocytes was determined in the presence of 12 µg/mL of either α-CR1 antibodies or IgY isotype control and expressed as the percentage of invasion into untreated (UT, no antibodies) control erythrocytes. Graphs represent the means ± standard errors of three independent experiment performed in duplicates. Statistical tests were performed using the Mann Whitney U test [p = 0.03 (*), p = 0.002 (**), p = 0.0002 (***), p < 0.0001 (****)].

Given the preference for SA-independent pathways across all isolates and the role of CR1 as a major receptor mediating this particular pathway, we sought to assess the contribution of CR1 in the invasion efficiency of Ghanaian clinical isolates. Overall, there was no significant effect of anti-CR1 antibodies on the parasites’ invasion efficiency into untreated erythrocytes. However, the invasion efficiency into neuraminidase treated erythrocytes was drastically reduced by the addition of anti-CR1 antibodies (mean, 22.67%) compared to the isotype control (IgY) (**Figure 4**). Across all isolates, the invasion rate into neuraminidase-treated erythrocytes ranged from 1.15 to 76.27% following the addition of anti-CR1 antibodies as compared to untreated erythrocytes. This further emphasizes the possible involvement of the PfRh4-CR1-mediated pathway in the observed SA-independent pathway. However, although invasion efficiency into neuraminidase-treated erythrocytes in the presence of anti-CR1 antibodies varied across isolates, these differences were only significant when comparing isolates from Kintampo and Navrongo (ANOVA P = 0.048; Tukey’s test P = 0.027).

### Expression levels of invasion ligands in Ghanaian *P. falciparum* clinical isolates

**Figure 5:**
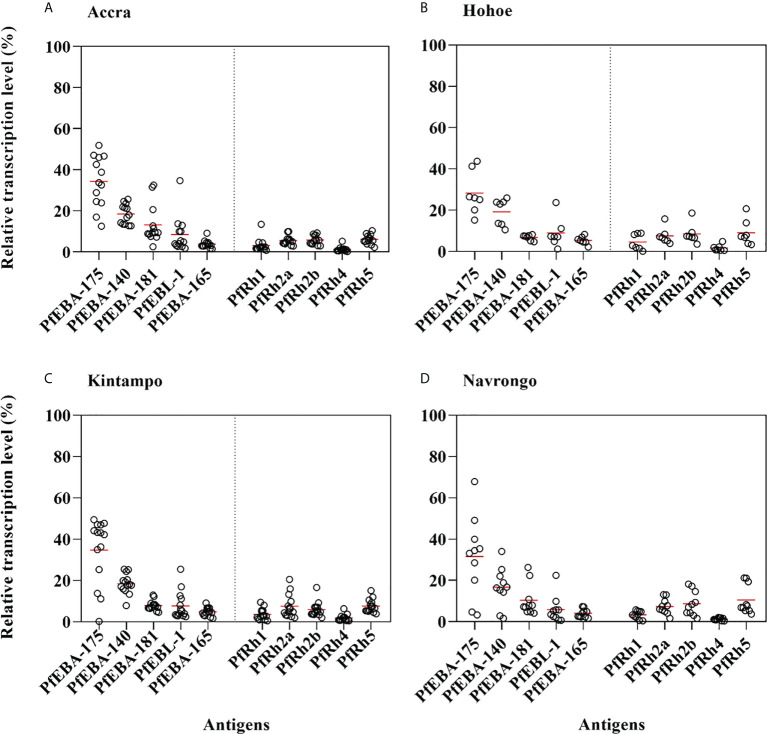
Relative transcript levels of invasion-related genes in Ghanaian *P. falciparum* clinical isolates. The transcript level of each gene was determined by RT-qPCR in 44 isolates from **(A)** Accra, N = 13; **(B)** Hohoe, N = 7; **(C)** Navrongo, N = 10; and **(D)** Kintampo, N = 14. The transcript level of each gene was expressed as a proportion of the total transcript level of the ten genes following normalization to that of the 60S ribosomal L18 protein and that of AMA1, respectively used as endogenous control and late-stage parasite marker.

**Figure 6:**
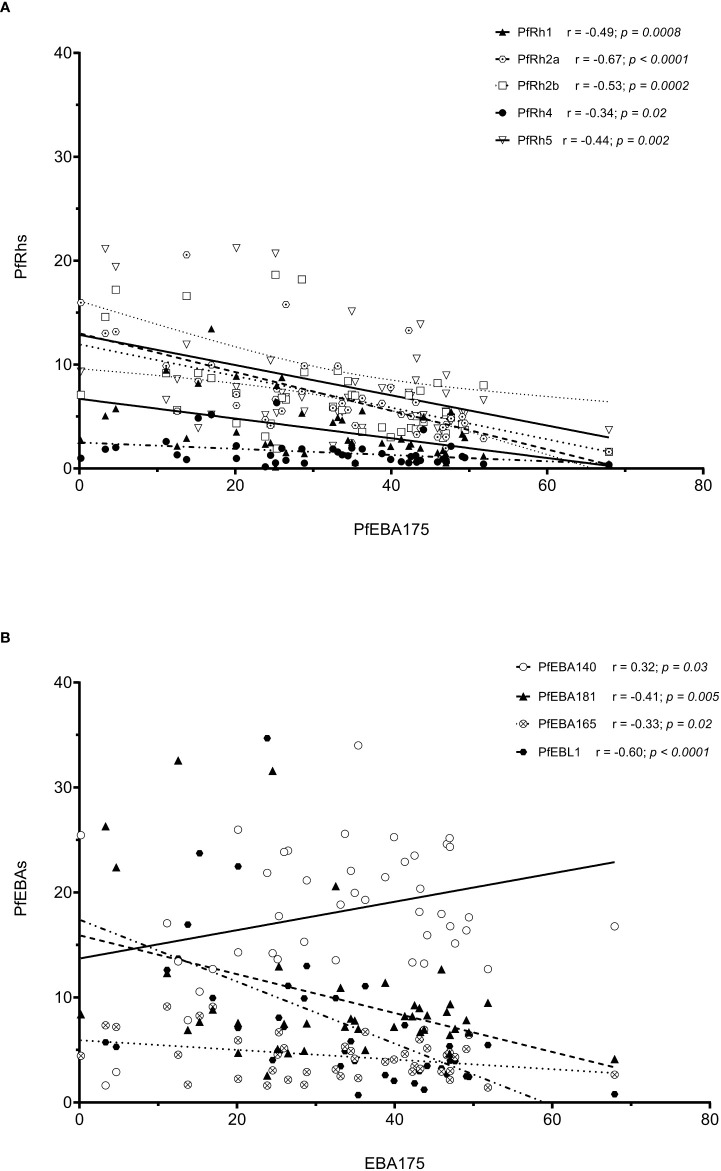
Correlation of the transcript level of EBA175 with other invasion related genes. Note: The high transcript level of EBA175 is associated with low levels of Rh genes **(A)**, while the transcript levels of EBA genes are negatively correlated with the transcript level of EBA175, except for EBA140, which show a moderated positive correlation with EBA175 **(B)**.

As erythrocyte invasion is driven by parasite antigens expressed or secreted to the surface of merozoites upon schizont maturation and egress, we sought to investigate the expression levels of the most characterized invasion-related genes over the last decades. Here, the gene expression analysis was performed for 10 different invasion-related genes (PfEBA175, PfEBA140, PfEBA181, PfEBA165, PfEBL1, PfRh1, PfRh2a, PfRh2b, PfRh4 and PfRh5) in a total of 44 isolates for which invasion data were also available (Accra = 13, Hohoe = 7, Kintampo = 14 and Navrongo = 10). Across all isolates, the expression of PfEBA genes was highly abundant compared to that of the PfRh genes (**Figure 5**). The pattern of gene expression was relatively similar for all sites with almost no difference in the relative expression of individual genes across all study sites (**Figure 5**). Although the expression levels of individual genes did not vary significantly across sites (**Figure S1**), there was a higher expression of PfEBA175 and PfEBA181 in isolates from Accra (mean, 34.68% and 13.2%, respectively), while isolates from Hohoe had the lowest transcript levels of the corresponding genes (mean, 28.29% and 6.80%, respectively). However, isolates from Hohoe presented the highest transcript levels for PfEBA165 (mean, 5.37%), and Rh1 (mean, 4.62.17%) (**Figure S1**). While the transcript level of PfRh2a appeared to be similar in isolates from Hohoe, Kintampo and Navrongo (mean, 7.51%, 7.51% and 7.37%, respectively), those of PfRh2b and PfRh5 were higher in isolates from Hohoe and Navrongo (**Figures 5**; **S1**). Furthermore, except for PfEBA140, there was a moderate to strong negative correlation between the expression level of PfEBA175, the most abundant transcript, and that of the rest of the ligands tested here (**Figure 6**).

## Discussion

*P. falciparum* clinical isolates used in this study were collected from Ghanaian children of age 2-14 years residing in four endemic areas. This age group constitutes a good cohort for understanding the dynamics of malaria transmission and the development of antimalarial immunity in exposed individuals. The data presented in this study showed that children from Kintampo and Navrongo were younger as compared to those from Accra and Hohoe. This trend confirms previous reports in Ghanaian children from areas of varying transmission (13, 22) and suggests that the age of vulnerability likely shifts higher in low transmission areas. Moreover, there was a significantly higher parasite density in children from Kintampo as compared to the rest of the study sites. This agrees with reports suggesting a higher threshold parasiteamia for symptomatic malaria in individuals from high transmission settings (13, 23).

In this study, *in vitro* invasion phenotyping data of 156 *P. falciparum* clinical isolates from four endemic areas in Ghana were compared and found to form a homogenous population in their use of the SA-independent invasion pathway. This study is the second reporting on the invasion phenotype of *P. falciparum* clinical isolates from different endemic areas within Ghana. Overall, there was a slight difference in the parasites’ sensitivity to neuraminidase treatment, which was only significant when comparing isolates from Kintampo and Navrongo; the two sites with the highest transmission intensity. This data differs from earlier reports from the Gambia, where the majority of isolates tested showed SA-dependent invasion phenotype (10). Our result also vary from previous reports from our group, which showed significant inverse relationship between sensitivity to neuraminidase treatment and transmission intensity (13). It however agrees with other findings from a collaborative team which reported no significant differences in the sensitivity of neuraminidase treatment when comparing isolates across different endemic countries (6). A plausible explanation for the discrepancies in results could be the differences in the number of isolates tested in each study. The earlier studies included 38 (10), 52 (13) and 67 (6) isolates respectively while ours involved the largest sample size (156 isolates), therefore providing higher analytical power. While no significant differences were observed in invasion efficiency upon treatment with chymotrypsin across all sites, there was significant differences invasion following treatment with trypsin when comparing isolates from Navrongo to those from Accra or Kintampo; once again contradicting previous results where no significant difference was observed in invasion efficiency across sites following trypsin treatment (6, 13). Again, these variations could be as a result of increased sample size in the current analysis, indicating the need for larger samples in determining patterns in invasion pathways in communities.

Furthermore, our data reveals the predominance of the NrTsCs invasion profile across all isolates, with strong positive correlation between sensitivity to trypsin and chymotrypsin treatment while neuraminidase sensitivity neither correlated with trypsin nor with chymotrypsin treatment. This confirms an earlier report from our group regarding the preference of SA-independent pathways in Ghanaian clinical isolates (13) and suggests that the majority of these isolates might be using alternative pathways mediated by receptors sensitive to both trypsin and chymotrypsin proteases. These data agree with the most recent reports on the field (6, 13), although older studies reported NrTsCr as the dominant profile in the Senegalese isolates (15), while NrTrCr was the most common profile found in Colombian and Peruvian isolates (21). These patterns could be driven by variations in transmission intensities as well as the clonality of the parasite populations in the area. Similar to previous reports (6), our data showed the absence of a relationship between the diversity of *P. falciparum* invasion profile and the malaria transmission intensity, with Accra and Kintampo representing the lowest and highest transmission settings, respectively, exhibiting the most homogenous parasite populations. However, the presence of certain invasion profiles (such as NrTrCs and NrTrCr) at specific sites (Hohoe and Navrongo) only, affirm the hypothesis for the selection of local parasite populations to maintain invasion phenotype diversity within a given area (6).

Given the strong positive correlation between sensitivity to trypsin and chymotrypsin treatment and the usage of SA-independent invasion pathway, we tested the impact of anti-CR1 on blocking invasion in the clinical isolates (13, 20, 24). The data confirmed previous findings that Ghanaian *P. falciparum* clinical isolates rely on the PfRh4-CR1 interaction as an alternative invasion pathway (13). Altogether, these data show that Ghanaian clinical isolates can use various pathways to achieve successful invasion. Consistent with previous findings, none of the receptors affected by the three enzymes is unanimously used by clinical isolates from a given area (6, 13, 15, 21). 

We also tested for the relative expression levels of known invasion-related genes and our data, once again, are in agreement with previous reports on Ghanaian clinical isolates (6, 13). There was a higher expression of PfEBA175 across all isolates (6, 13, 25). Also a strong negative correlation was observed between the expression levels of PfEBA175 and PfRh4 in this study, similarly to earlier reports (13). The higher transcript level of PfEBA175 in isolates from Accra, although not significant, is in agreement with suggestions regarding the use of this ligand in non-immune individuals (6), since Accra represents the lowest transmission setting.

Of particular interest here was the high expression levels of PfRh2b and PfRh5 in isolates from Hohoe and Navrongo, which also happened to host the parasites populations with the highest invasion diversity. Of the two invasion profiles unique to Hohoe and Navrongo, NrTrCs and NrTrCr, the later is typical to the previoulsy reported PfRh5-Basigin mediated pathways (26–28). On the other hand, PfRh2b binding to its so far unknown erythroctye partner has been shown to be neuraminidase and trypsin resistant (29). Elevated expression of Rh2b has also been linked to invasion phenotype switching (PMCID: PMC6289454; PMCID: PMC6959351), with a chymotrypsin sensitive phenotype favoured (PMCID: PMC6289454). Again, the interaction between the processed fragment of PfRh2b and its erythrocyte partner has been shown to facilitate the PfEBA175-GYPA interaction during invasion through a Ca^2+^ signaling process (30). This interaction has earlier been shown to be prevented in a dose-dependent manner by erythrocyte treatment with chymotrypsin, peaking at 2 mg/mL enzyme concentration (30). We however used 1 mg/mL enzyme concentration in the current study, based on hindsight knowledge that this concentration is sufficient for efficient erythrocyte surface receptor removal (PMCID: PMC4577045) leaving room for possible downstream signaling processes leading to successful invasion. We used 1 mg/mL of chymotrypsin in the present study based on previous knowledge that higher concentrations yielded similar levels of efficiency. Moreover, we recently showed that the efficiency of enzyme treatment could be influenced by erythrocytes’ surface receptor densities (31). Notwithstanding, the current observation about Rh2b, together with previous data, highlight the contributory role of Rh2b to erythrocyte invasion and its likely involvement in dictating phenotypic variation.

Additionally the level of immunity in the general population could influence the selection of the different ligands driving the different invasion pathways. Immunity has been hypothsized to influence the deletion of PfRh2b -specific region (32). Though not independently verified in Ghana, PfRh2b-specific region deletion has been characteriszed among Ghanaian parasite isolates (33), suggesting an attempt to use alternate pathways not driven by this antigen.

To sum up, our findings indicate that Ghanaian *P. falciparum* clinical isolates predominantly invade erythrocytes through a SA-independent pathway (CR1-mediated pathway) irrespective of the level of transmission intensity. Additionally, across all isolates, the NrTsCs was the most predominant profile, suggesting a major contribution of the PfRh4-CR1 interaction during the invasion of Ghanaian clinical isolates.

## Data availability statement

The original contributions presented in the study are included in the article/**Supplementary Materials** further inquiries can be directed to the corresponding authors.

## Ethics statement

The study was approved by the Institutional Review Board of the Noguchi Memorial Institute for Medical Research, University of Ghana (IRB00001276) and the Ghana Health Service Ethical Review Committee (GHC-ERC: 005/12/2017). Written informed consent to participate in this study was provided by the participants’ legal guardian/next of kin.

## Author contributions

LT, YA, and GA conceived the study; LT, PN, and FA performed the experiments LT, PN, FA, MN, YA, and GA analyzed the data and drafted the manuscript; YA, GA, and MN supervised the study. All authors critically reviewed and edited the manuscript.

## Funding

This work was supported by funds from a World Bank African Centres of Excellence grant (ACE02-WACCBIP: Awandare) and a DELTAS Africa grant (DEL-15-007: Awandare). LT was supported by WACCBIP-World Bank ACE PhD fellowships, respectively, FA was supported by the National Institute for Health Research (NIHR) Global Health Research program 16/136/33, using aid from the UK Government while YA was supported by a Crick African Network which receives its funding from the UK’s Global Challenges Research Fund (MR/P028071/1), and by the Francis Crick Institute which receives its core funding from Cancer Research UK (FC1001647), the UK Medical Research Council (FC1001647), and the Wellcome Trust (FC1001647). The DELTAS Africa Initiative is an independent funding scheme of the African Academy of Sciences (AAS)’s Alliance for Accelerating Excellence in Science in Africa (AESA) and supported by the New Partnership for Africa’s Development Planning and Coordinating Agency (NEPAD Agency) with funding from the Wellcome Trust (107755/Z/15/Z: Awandare) and the UK government. The views expressed in this publication are those of the author(s) and not necessarily those of AAS, NEPAD Agency, Wellcome Trust or the UK government.

## Acknowledgments

The authors are grateful for the technical support of the members of WAMIN and the Molecular genetics and Cell Biology Research group at WACCBIP for the scientific feedback on the execution of this project.

## Conflict of interest

The authors declare that the research was conducted in the absence of any commercial or financial relationships that could be construed as a potential conflict of interest.

## Publisher’s note

All claims expressed in this article are solely those of the authors and do not necessarily represent those of their affiliated organizations, or those of the publisher, the editors and the reviewers. Any product that may be evaluated in this article, or claim that may be made by its manufacturer, is not guaranteed or endorsed by the publisher.

## Supplementary material

Supplementary Figure 1 Relative transcript levels of individual invasion-related genes in *P. falciparum* clinical isolates across the various study site. The transcript level of each gene was determined by RT-qPCR in 44 isolates from **(A)** Accra, N = 13; **(B)** Hohoe, N=7; **(C)** Navrongo, N = 10; and **(D)** Kintampo, N = 14. The transcript level of each gene was expressed as a proportion of the total transcript level of the ten genes following normalization to that of the 60S ribosomal L18 protein and that of AMA1, respectively used as endogenous control and late-stage parasite marker. The Kruskal-Wallis test was used to test for differences in the gene transcript levels across the various sites showed no statistical difference in the expression level of individual genes.
