# Supplementary figures and images for: Phenotypic characterization of Ghanaian P. falciparum clinical isolates reveals a homogenous parasite population
Source: Front Immunol. 2022 Sep 23;13:1009252. doi: 10.3389/fimmu.2022.1009252 (PMC9537689; doi:10.3389/fimmu.2022.1009252)

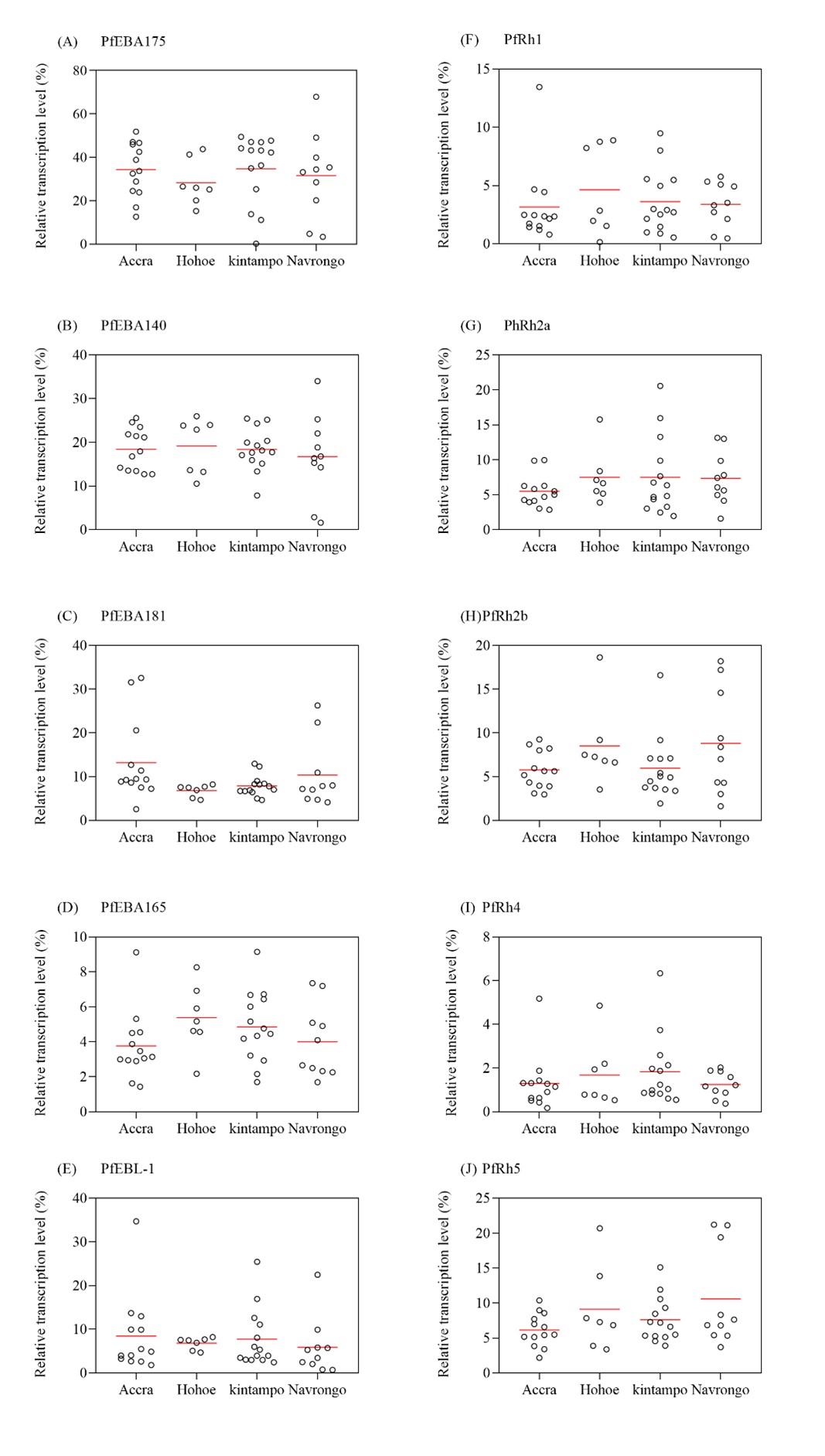

Supplement: Supplementary Figure 1 — Relative transcript levels of individual invasion-related genes in P. falciparum clinical isolates across the various study site. The transcript level of each gene was determined by RT-qPCR in 44 isolates from (A) Accra, N = 13; (B) Hohoe, N=7; (C) Navrongo, N = 10; and (D) Kintampo, N = 14. The transcript level of each gene was expressed as a proportion of the total transcript level of the ten genes following normalization to that of the 60S ribosomal L18 protein and that of AMA1, respectively used as endogenous control and late-stage parasite marker. The Kruskal-Wallis test was used to test for differences in the gene transcript levels across the various sites showed no statistical difference in the expression level of individual genes. [file Image_1.jpeg]
